# Risks and chances of aberrant DNA repair in cancer

**DOI:** 10.18632/oncoscience.459

**Published:** 2018-08-22

**Authors:** Maria Schubert, Richard Greil, Roland Geisberger

**Affiliations:** Department of Internal Medicine III with Haematology, Medical Oncology, Haemostaseology, Infectiology and Rheumatology, Oncologic Center, Paracelsus Medical University, Salzburg, Austria; Salzburg Cancer Research Institute - Laboratory for Immunological and Molecular Cancer Research (SCRI-LIMCR), Cancer Cluster, Salzburg, Austria

**Keywords:** DNA repair, chromosomal rearrangements, cancer, leukemia, therapy

Genomic aberrations, such as translocations, deletions or more complex “chained” rearrangements (chromotrypsis, chromoplexis) of DNA are frequently found in almost any cancer entity. These rearrangements can be clonal and stable during the course of disease – thus pointing to an initializing event – or subclonal, with specific rearrangements being present in specific fractions of cancer cells with fraction sizes varying during disease progression. In the latter scenario, genomic instability of the cancer genome results in longitudinal subclonal diversification during disease progression, contributing to high clonal dynamics and finally to the selection of treatment refractory clones and disease relapse. While genome rearrangements involving loss or amplification of cancer related genes are apparent drivers that give a growth advantage to the cell, also balanced rearrangements without loss of genetic material can contribute to clonal fitness by generating fusion proteins or by affecting the expression of genes adjacent to breakpoint junctions. Particularly in chronic lymphocytic leukemia, complex karyotypes (defined as presence of at least three chromosomal aberrations) often go along with bad prognosis and poor response to therapies. Resistance to novel Bruton’s Tyrosine kinase (BTK) inhibitors (ibrutinib) or BCL2 inhibitors (venetoclax) is more frequent in cases with complex karyotypes [1, 2]. Ibrutinib resistance is mostly due to specific mutation of BTK or phospholipase gamma downstream of BTK, and these mutations frequently lead to a Richter transformation from chronic lymphocytic leukemia to diffuse large B cell lymphoma. While Richter transformed leukemia is refractory to ibrutinib, it surprisingly becomes more sensitive to immune checkpoint inhibitors (therapeutic PD-1 or PD- L1 antibodies, which aim at reinvigorating anti-cancer T cell immunity) [3]. The fact that BTK mutations in CLL frequently coincide with complex karyotypes could mean that some cellular processes, such as replication stress or DNA damage by reactive metabolites or treatment, increase both, mutations due to error prone repair mechanisms as well as structural variations by aberrant and imprecise end joining. In addition, mutation rates can be increased during end joining by AID/APOBEC deaminases that induce mutations flanking the DNA ends prior repair and by error prone DNA polymerases [4]. Eventually, this increase in mutations and structural variations not only enhances aggressiveness of cancer cells, but may also lead to increased tumor antigen load, enforcing cancer immune interactions, thus, sensitizing for immune checkpoint therapies [5]. In our own work, we recently showed that leukemic cells have an imprecise repair of DNA double strand breaks, characterized by increased joining of incompatible DNA ends due to a bias towards microhomology mediated end joining (MMEJ) [6]. In contrast to classical non- homologous end joining (NHEJ, direct joining of DNA ends or usage of short <5 nt homologies), MMEJ uses larger regions of homologous DNA for joining of distant DNA ends and is more error prone than NHEJ. As microhomologies are frequently found at breakpoints from chromosomal rearrangements in cancer genomes, this repair pathway significantly contributes to the acquisition of chromosomal rearrangements, facilitating novel subclonal rearrangements and hence, fueling clonal evolution [7]. Cancers with DNA repair defects are often sensitive to inhibition of remaining repair pathways (synthetic lethality), and hence, it is conceivable that also aberrant end joining in leukemia could be harnessed for treatment [8]. In this regard, an intrinsically increased error prone DNA end joining could further increase chromosomal rearrangements upon treatment with DNA damaging agents (eg with DNA intercalating drugs or targeting of factors of homologous recombination) and thus, increasing tumor antigen load and sensitizing for immune checkpoint inhibition similar to CLL patients with Richter transformation. Conversely, concomitant targeted inhibition of MMEJ factors could impede clonal evolution during conventional treatment, thus counteracting and deferring relapse and drug resistance (Figure 1). Summarizing, while DNA repair defects or biased DNA repair enable cancer cells to diversify their genetic material during disease progression, they also provide a basis for novel treatment options, warranting further research on DNA repair in context of cancer.

**Figure 1:**
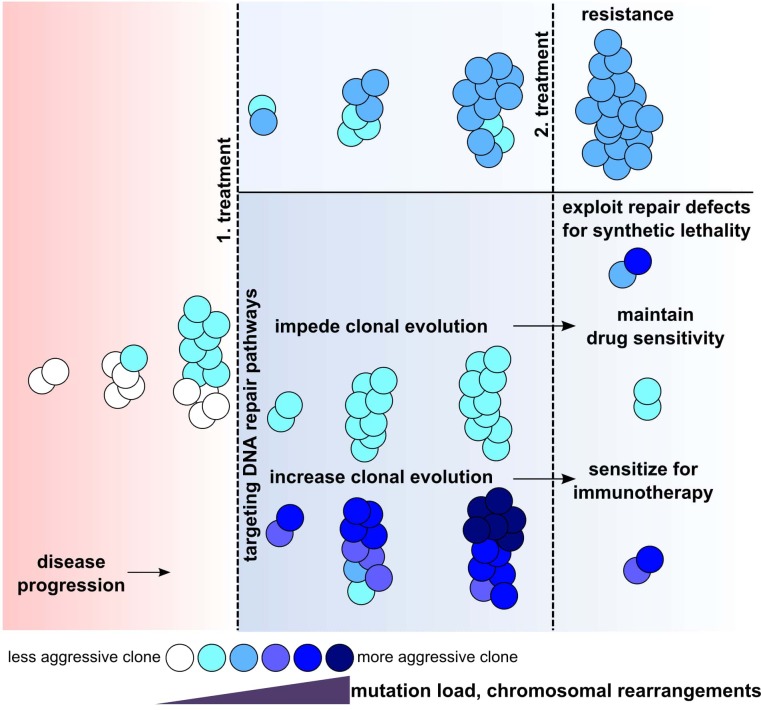
DNA repair in context of cancer progression and possible outcomes upon targeted inhibition of distinct DNA repair pathways

## References

[1] Taylor BJ (2013). Elife.

[2] Anderson MA (2017). Blood.

[3] Ding W (2017). Blood.

[4] Taylor BJ (2013). Elife.

[5] Rizvi NA (2015). Science.

[6] Gassner FJ (2018). Mol Cancer Res.

[7] McVey M (2008). Trends Genet.

[8] Percy SI (2016). Trends Cancer.

